# Optimized production of laccase from *Pseudomonas stutzeri* and its biodegradation of lignin in biomass

**DOI:** 10.1007/s12223-024-01232-6

**Published:** 2024-12-11

**Authors:** Waqar Rasool Minhas, Saira Bashir, Cheng Zhang, Ahmad Raza

**Affiliations:** 1 National Institute for Biotechnology and Genetic Engineering (NIBGE), Jhang Road, P.O. Box 577, Faisalabad, Pakistan; 2 Pakistan Institute of Engineering and Applied Sciences (PIEAS), Islamabad, Pakistan; 3 Department of Chemistry, Biochemistry and Physics, South Dakota State University, Brookings, SD USA

**Keywords:** Laccase, Biodegradation, Biomass, Response surface methodology, Enzyme activity, Lignin

## Abstract

Laccases are multi-copper oxidases that play an important role in the biodegradation of phenolic compounds, lignin, dye, and wastes. Here, we report the screening of potential laccase-producing indigenous bacterial isolates and subsequent optimization of laccase production using crop residues as cheap supplementary energy sources. Among 16 bacterial isolates, seven were selected based on the appearance of reddish-brown bacterial colonies and guaiacol oxidation assay after 10 days of incubation at 37 °C. The maximum laccase activity (2.755 U/mL) was observed for bacterial isolate WR2. Response surface methodology (RSM) was used to maximize laccase production from WR2, identified as *Pseudomonas stutzeri*. Plackett–Burman design (PBD) was employed to design production runs involving various factors including time, pH, inoculum, wheat straw, cotton stalk, wheat bran, rice straw, copper sulfate, sugarcane bagasse, yeast extract, and peptone. The interactions of different factors were analyzed from the responses (laccase enzyme activity, etc.) in 12 experimental runs. In experimental run 4, the maximum laccase enzymatic activity (1.86 U/mL) was achieved after a 10-day incubation with wheat straw (1%) and cotton stalk (1%) at pH 6.8 and 37 °C, and high-degree lignin degradation was evident from a substantial reduction in the FTIR aromatic stretching peak of the degraded biomass.

## Introduction

Among lignin-degrading enzymes including laccase, manganese peroxidase, lignin peroxidase, and several H_2_O_2_-forming oxidases (Kirk and Farrell 1987), laccase enzymes play a more important role in lignin degradation. Laccase is the oldest and most studied enzyme. It belongs to a multi-copper protein family that constitutes ceruloplasmin, ascorbate oxidase, and bilirubin oxidase (Hoegger et al. 2006), and a copper atom is essential to their catalytic activity (Quintanar et al. 2005). Laccase is formed by higher plants, bacteria, and fungi (Benfield et al. 1964). Many fungi secrete laccases extracellularly as a secondary metabolite. In addition to lignin, laccases can catalyze oxidative degradation of a wide range of substrates including methoxy-substituted phenols, ortho- and para-diphenols, phenolic acids, and non-phenolic compounds (Atalla et al. 2013; Hakulinen and Rouvinen 2015) via a radical mechanism (Banci et al. 1999).

Efficient isolation and characterization of potent lignin-degrading microbes and enzymes will have a great impact on the biofuel and paper pulp industry, as well as the decolorization of dye and biodegradation/detoxification of wastes. Qualitative and quantitative methods for isolation and characterization of lignin-modifying enzymes secreted by microorganisms have been reviewed by Kameshwar and Qin (2017). After isolation of the microbes, optimization of their enzyme production conditions is essential for efficient production of laccases. Submerged fermentation is the method by which maximum laccase production can be attained (Singh et al. 2009). Screening of parameters such as temperature, different concentrations of yeast extract, magnesium sulfate, and time of incubation using response surface methodology (RSM) has been done in literature for laccase production by *Streptomyces psammoticus* (Niladevi et al. 2009). A similar type of study was done for the production of laccase at a temperature of 37 °C from *Bacillus cereus* using RSM with Box-Behnken design (Ding et al. 2012; Rajeswari et al. 2015).

In this study, optimization of laccase production is performed on the following factors: pH, yeast extract, peptone, CuSO_4_, sugarcane bagasse, wheat bran, wheat straw, rice straw, and cotton stalk. pH plays a vital role in changing the ionization state of the laccase enzyme (Reiss et al. 2013). CuSO_4_ is known to have a very potent effect as an inducer for laccase production as described by Gomaa and Momtaz (2015). Yeast extract and peptone are commonly used as nutrients for bacterial culture media. Different types of lignin have been utilized as a fermentation substrate to produce lignin-modifying enzymes and other value-added products (Iram et al. 2021). Very recently, laccase production from sugarcane bagasse, reed grass, rice bran, wheat bran, wheat straw, corn cobs, guava leaves, and peanut husk using solid-state fermentation was reported (Hasan et al. 2023). The highest level of laccase activity, 2.551 U/mL, was produced from wheat straw. The novelty of the research is to use crop residues such as wheat straw, wheat bran, cotton stalk, and sugarcane bagasse as cheap supplementary energy sources for microorganisms to produce laccase. The experiments were designed, and the results were analyzed following RSM. A guaiacol oxidation assay was performed to monitor laccase productivity, and FTIR analysis was done on the most productive run to verify the action of laccase enzyme on lignocellulosic biomass during laccase production.

## Materials and methods

A total of five soil samples were collected from a solid waste site at Jaranwala Road, Muhammad Wala, Faisalabad. Two soil samples were selected from the five soil samples by observation of the decay of wood and leaf.

### Bacterial isolate screening and selection and laccase production

To isolate the bacteria, 1 g of the soil sample and 9 mL of autoclaved distilled water were gently mixed. The mixture was diluted by a factor of 10^10^ to be used as inoculum for bacterial growth in autoclaved nutrient agar plates using the spread plate technique. Each agar plate contained 20 mL of a medium made from 3 g of a Merck nutrient agar mixture (peptone 0.5%, beef extract/yeast extract 0.3%, agar 1.5%, NaCl 0.5%) and 100 mL of distilled water. The plates were placed in a Thelco incubator, set at 30 °C, for 1 day. Different bacterial strains were identified and isolated based on morphology, texture, and color.

To screen the bacterial isolates with potential for laccase production, all the isolates were cultured on autoclaved nutrient agar guaiacol (NAG) plates using the spread plate technique. Each NAG plate contained 20 mL of a medium made from 3 g of a nutrient agar mixture from Merck (peptone 0.5%, beef extract/yeast extract 0.3%, agar 1.5%, NaCl 0.5%), 50 mL of 0.5 mM guaiacol, and water (to make the total volume of medium 100 mL). The pH of the medium was adjusted to 6.8 using diluted HCl and NaOH (a few drops). After the addition of bacterial isolates, all the NAG plates were placed in a Thelco incubator, set at 30 °C, for 7 days. All the plates were observed on a daily basis for the appearance of reddish-brown colonies. Those colonies which showed brown color were considered as potential isolates for the production of laccase. Seven bacterial isolates (WR1, WR2, WR3, WR4, WR5, 13P*, and 38R) were identified as potential laccase producers.

To study laccase enzyme productivity of the seven bacterial isolates, a similar amount of bacterial isolate was taken from each NAG plate and added to an Erlenmeyer flask with autoclaved 100 mL nutrient broth prepared from 3 g of nutrient mixture from Merck (peptone 0.5%, beef extract/yeast extract 0.3%, NaCl 0.5%), 50 mL of 0.5 mM guaiacol, and distilled water (to make the total volume of broth 100 mL). The flasks are placed in a shaking incubator at 37 °C and 145 rpm and were observed on a daily basis for growth and color change.

Laccase enzyme activity in μmol/(min mL) or U/mL is defined as the amount of enzyme required to oxidize 1 μmol of guaiacol per minute per unit volume. It was quantitatively determined by guaiacol oxidation assay (i.e., laccase assay) performed for each broth every day during the course of 10 days following the protocol described below.

Aliquot (1 mL) was taken from a broth, centrifuged to remove solid, and then mixed with guaiacol (2 mM, 1 mL) and sodium acetate (10 mM, 3 mL) in a vial. The mixture was incubated in a water bath at 30 °C for 5 min. The absorbance was taken at 450 nm for these mixtures (termed “laccase assay mixtures”) using a spectrophotometer. Enzyme activity was then calculated using the formula below, which can be derived from the Lambert–Beer law. This assay was performed in triplicate. A blank was also prepared using 1 mL of distilled water instead of broth aliquot.

$$\text{L}.\text{A}.=A\times V/\left(e\times l\times t\times v\right)$$

where L.A. is the laccase activity in μmol/(min mL) or U/mL, *A* is the absorbance, *V* is the total volume of laccase assay mixture in liters (0.005 L), *e* is the extinction coefficient for guaiacol (0.6740 μM^−1^ cm^−1^), *l* is the light path length in cm (1 cm), *t* is the incubation time in minutes, and *v* is the enzyme volume in mL (1 mL).

Gene sequencing was used for bacterial identification. The genomic DNA was isolated from the bacterial isolates. The DNA purity was checked by measuring the *A*_260_/*A*_280_ ratio, which is 1.8, and then, this DNA was selected for the PCR amplification. Among the various gene targets, the 16S ribosomal DNA (rRNA) gene is the most common target used (Lau et al. 2015). To target the 16S rRNA gene, primers for conserved regions were used. PCR product of 1500 pb was purified by using a PCR purification kit (Fermenta). The purified PCR products were sequenced by Eurofins, USA.

## Results and discussions

### Screening of the potential bacterial isolates

Bacterial growth was performed for diluted water extracts of two soil samples using the spread plate method in nutrient agar plates. A total of 16 bacterial strains were identified and isolated based on morphology, texture, and color. These colonies were isolated and screening of these isolates was done in nutrient agar plates containing guaiacol as differential media. Potential laccase-producer bacterial isolates changed the color of the colony to reddish-brown after 10 days at 37 °C as presented in Table 1. The color change is an indicator of the presence of laccases, which catalyze guaiacol oxidation to produce light absorption at 460 nm (Vignesh et al. 2016). The seven potential laccase-producing bacterial strains (WR1, WR2, WR3, WR4, WR5, 13P*, and 38R) were identified. Gram staining was done for the bacterial isolates on glass slides, and the shape of the colonies was observed under a 100 × microscope. Their color, elevation, texture, margins, and shapes are elaborated in Table 1.

**Table 1:**
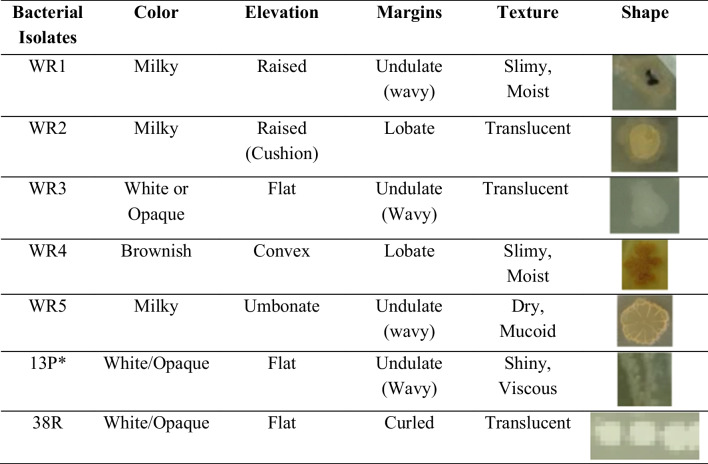
Morphological characteristics of isolated bacterial colonies

#### Catalase test

H_2_O_2_ drops were put on each isolate smear on microscopic glass slides, and observations were made on bubble formation. Negative catalase responses (few or no bubbles) were observed for WR1, WR3, and WR4, while positive catalase responses were observed for WR2, WR5, 38R (bubble formations), and 13P* (excellent bubble formation).

#### Growth on MacConkey agar

Seven bacterial isolates were streaked on MacConkey agar plates and the plates were incubated overnight at 37 °C. MacConkey agar is a selective and differentiating agar that only grows Gram-negative bacterial species. Results showed that all seven bacterial isolates were Gram negative.

### Production of laccase enzyme

#### Study of laccase enzyme productivity in nutrient broth

To study laccase enzyme productivity, the seven bacterial isolates were grown in nutrient broth (NB) containing 0.5 mM of guaiacol and laccase activity was checked on a daily basis. Gradual changes in the color of media from yellow to brown were observed during laccase production, showing laccase’s ability to oxidize guaiacol. Guaiacol oxidation in the broth was monitored by optical density measurement of aliquots taken from the broths. Laccase enzyme activity in μmol/(min mL) or U/mL, defined as the amount of enzyme required to oxidize 1 μmol of guaiacol per minute per unit volume, was quantitatively determined by a guaiacol oxidation assay (i.e., laccase assay) performed for each broth every day during the course of 10 days following the protocol described in the “Materials and methods” section.

**Fig. 1:**
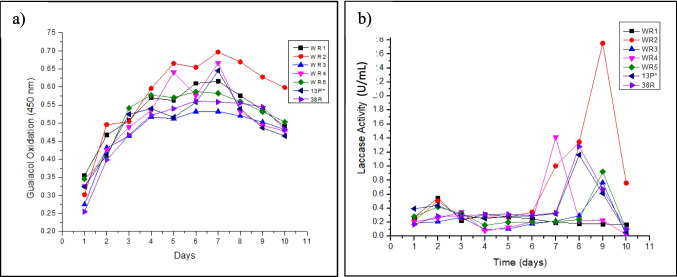
**a** Optical density at 450 nm of laccase assay mixtures of selected bacterial isolates. **b** Laccase activity of selected bacterial isolates

The results of optical density measurement and laccase activity calculation are shown in Fig. 1a, b. The highest optical density of the guaicol oxidation product was observed for WR2 on day 7. Later, the optical density decreased with time, indicating that the guaiacol oxidation product was not stable in the broth. This justifies a separate laccase activity assay that was performed, where fresh guaiacol was added and the mixture was only incubated for a short time to minimize further degradation of the guaiacol oxidation product. The result in Fig. 1b shows that the maximum laccase activity (2.755 U/mL) was observed by the potential WR2 isolate on day 9 while the lowest activity was observed by the WR1 isolate.

#### Molecular phylogenetic analysis by maximum likelihood method

**Fig. 2:**
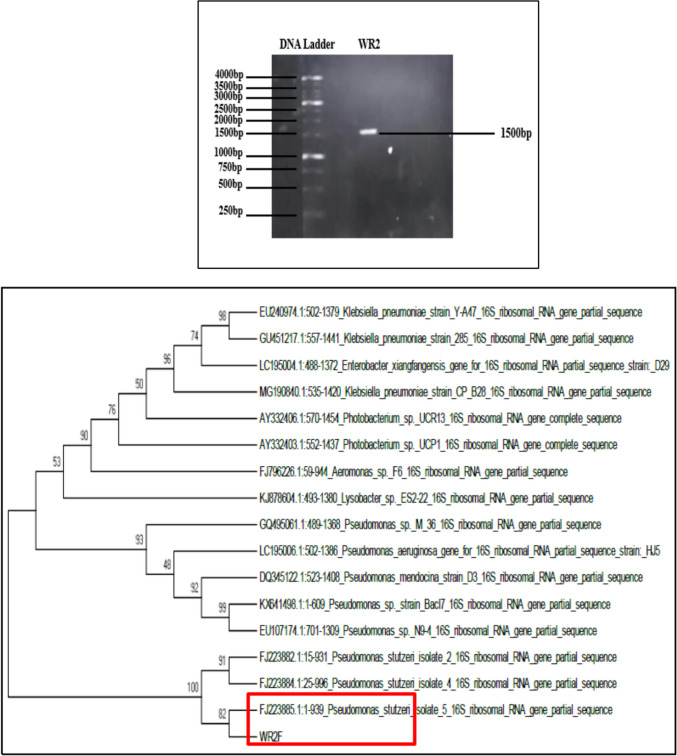
16S rRNA gene 1500bp of WR2

The WR2 isolate was identified as *Pseudomonas stutzeri* (Fig. 2) using NCBI BLAST of 16S rRNA gene sequences and its maximum similarity was 99.89%. Different strains of this bacterium have been reported for their laccase production for biodecolorization of textile azo dyes (Bera and Tank 2021; Joshi et al. 2020; Kuppusamy et al. 2017), use as a green catalyst (Shinde et al. 2020), crude oil degradation (Parthipan et al. 2017), etc.

### RSM study of laccase production using lignocellulosic biomass

To maximize the production of laccase from bacterial isolate WR2 using lignocellulosic biomass, 12 runs of experiments of Plackett–Burman design (Table 2) were performed on the following growth parameters: pH (5.5 or 6.8), incubation time (5 or 10 days), inoculum (1 or 2%), sugarcane bagasse (0 or 1%), wheat bran (0 or 1%), wheat straw (0 or 1%), rice straw (0 or 1%), cotton stalk (0 or 1%), yeast extract (0 or 0.5%), peptone (0 or 0.5%), and CuSO_4_ (0 or 100 µM). All the percentages were based on the amount of autoclaved distilled water (100 g) used to make the broth. Laccase inoculum was added after each parameter takes only two values as shown in the parentheses.

The observed laccase activities are given in the last column of Table 2. The highest laccase activity (1.86 U/mL) was achieved in run 4 under the following conditions: pH 6.8, 10 days, inoculum 1 mL/100 mL, sugarcane bagasse 0%, wheat bran 0%, rice straw 0%, wheat straw 1%, cotton stalk 1%, yeast extract 0.5%, peptone 0%, and CuSO_4_ 100 µM, while the lowest laccase activity (0.086 U/mL) was observed in run 2 under the following conditions: pH 5.5, 5 days, inoculum 1 mL/100 mL, sugarcane bagasse 0%, wheat bran 0%, rice straw 0%, wheat straw 0%, cotton stalk 0%, yeast extract 0%, peptone 0%, and CuSO_4_ 0 µM.

**Table 2:** Twelve Plackett–Burman designs and observed laccase activity

Run	pH	Time (days)	Inoculum	Sugarcane bagasse	Wheat bran	Wheat straw	Rice straw	Cotton stalk	Yeast extract	Peptone	CuSO_4_ (µM)	Laccase activity
1	6.8	10	2	0	0	0	1	0	0.5	0.5	0	0.67
2	5.5	5	1	0	0	0	0	0	0	0	0	0.086
3	5.5	5	1	1	0	1	1	0	0.5	0.5	100	0.26
4	6.8	10	1	0	0	1	0	1	0.5	0	100	1.86
5	6.8	5	1	0	1	0	1	1	0	0.5	100	0.46
6	5.5	5	2	0	1	1	0	1	0.5	0.5	0	0.29
7	6.8	5	2	1	0	1	1	1	0	0	0	0.57
8	5.5	10	1	1	1	0	1	1	0.5	0	0	0.61
9	5.5	10	2	0	1	1	1	0	0	0	100	0.87
10	5.5	10	2	1	0	0	0	1	0	0.5	100	0.33
11	6.8	5	2	1	1	0	0	0	0.5	0	100	0.28
12	6.8	10	1	1	1	1	0	0	0	0.5	0	0.90

The data was statistically analyzed using Design Expert 10.0.1.0 to obtain the coefficient of determination (*R*^2^), which is a statistical measure of the dependence of laccase activity on the value of a growth parameter. With the *R*^2^ estimates obtained (Table 3), the dependence of laccase activity on growth parameters can be expressed in the formula below.

**Table 3:** Statistical summary of response surface methodology for the production of laccase activity

Factor	*R*^2^ estimate
*A*—pH	0.19
*B*—Time	0.27
*C*—Inoculum	− 0.097
*D*—Sugarcane bagasse	− 0.11
*E*—Wheat bran	− 0.032
*F*—Wheat straw	0.19
*G*—Rice straw	− 0.025
*H*—Cotton stalk	0.087
*J*—Yeast extract	0.062
*K*—Peptone	− 0.11
*L*—CuSO_4_	0.078

$$\mathrm{Laccase}\;\mathrm{activity}\;=\;0.60\;+\;0.19A+0.27B-0.097C\mathit-0.11D\mathit-0.032E+0.19F\mathit-0.025G+0.087H+0.0621I-0.11\;J+0.078\;K\\$$

where variables *A* through *K* are the factors listed in Table 3. The negative sign in the equation shows a negative effect while the positive sign shows a positive effect. The formula can be used to estimate laccase activity for different production conditions using different values (within the scopes of values used in the experiments as given in Table 2) for the variables.

**Fig. 3:**
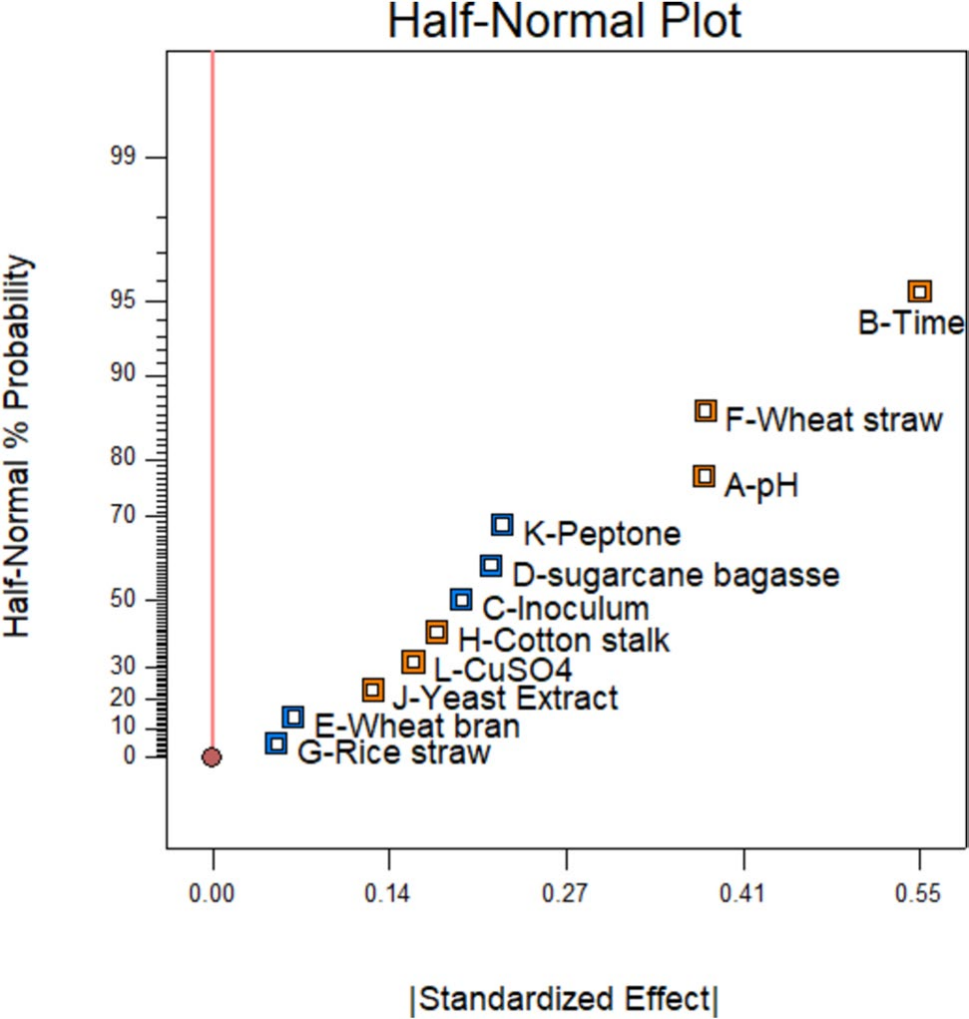
Half-normal plot with % probability of individual factors

A half-normal plot is presented in Fig. 3 to visualize the importance of the various factors, where the orange color presents a positive effect while the blue color presents a negative effect. Time, pH, and wheat straw are the factors with the highest probability of positive effects.

**Fig. 4:**
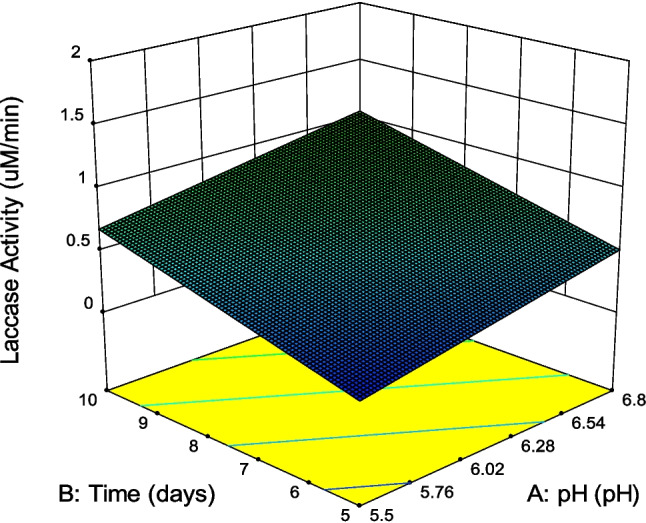
Response surface curves (3D plots) showing response of laccase to both time and pH

3D response surface graphs can be plotted to show the effect of two variables on laccase activity. Figure 4 shows the effect of time and pH on laccase activity which highlighted that time and pH changed while other factors remained constant. An increase in time resulted in increased laccase activity whereas the same trend has also been observed with pH and wheat straw. Similar 3D response surface graphs can be obtained for various pairs of positive factors such as pH/wheat straw, pH/cotton stalk, and pH/CuSO_4_.

### Fourier transform infrared spectroscopy (FTIR)

**Fig. 5:**
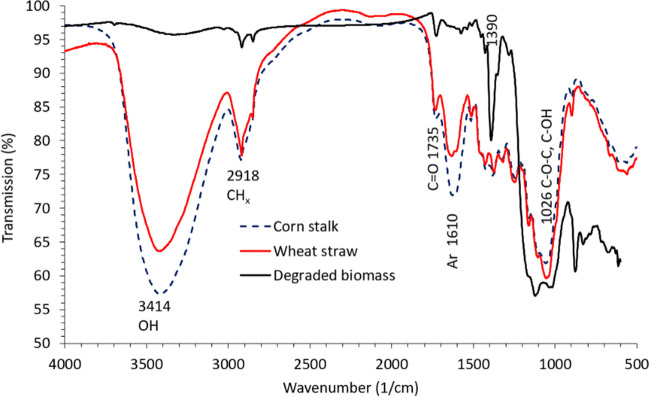
Fourier transform infrared spectrum (FTIR) showing structural changes in biomass from run 4

FTIR spectra of the biodegraded wheat straw and cotton stalk in run 4 are given in Fig. 5, together with the spectra of pristine wheat straw and cotton stalk. The pristine biomass spectra are very similar to the spectra in the literature (Adapa et al. 2011; Zeng et al. 2011). One of the most prominent changes is the weakening of the aromatic vibration peak at 1610 cm^−1^ and bending vibration peaks of Ar–H in the area of 1400–1300 cm^−1^, clearly indicating the substantial removal of lignin from the biomass by laccase. Reduction in this peak (at a lesser degree) has been reported as an indication of lignin degradation in wheat straw by lignin peroxidase expressed by *Phanerochaete chrysosporium* (Zeng et al. 2011). The strong peak at 1390 cm^−1^ and enhancement and broadening of the C-O signal at 1026 cm^−1^ are likely due to the inclusion of solid impurities which cannot be separated from the degraded biomass.

## Summary and conclusion

Seven potential laccase-producing bacterial isolates (WR1, WR2, WR3, WR4, WR5, 13P*, and 38R) were screened out by microscopic examination, biochemical assays, and cultivation on a guaiacol agar plate. It was found that the WR2 isolate showed maximum laccase enzymatic activity (2.755 U/mL) after 10 days of incubation at 37 °C in guaiacol-added nutrient broth. Guaiacol oxidation OD at 450 nm and cell culture OD at 600 nm were monitored on a daily basis for 10 days. Maximum guaiacol oxidation was observed on day 7 for WR2. By phylogenetic tree analysis, the WR2 isolate was identified as *Pseudomonas stutzeri* using NCBI BLAST of 16S rRNA gene sequences; the genetic hierarchy of WR2 was 99.89% similar to *Pseudomonas stutzeri*.

The Plackett–Burman design of the response surface methodology (RSM) was employed to investigate the effect of various physical and nutritive parameters such as pH, inoculum, time, wheat straw, wheat bran, rice straw, cotton stalk, sugarcane bagasse, yeast extract, peptone, and copper sulfate on laccase enzyme production. Among 12 experimental runs (PBD), maximum laccase production (1.86 U/mL) was observed in experimental run 4 by bacterial isolate WR2, while the minimum laccase activity was observed in experimental run 2 (0.086 U/mL) in submerged fermentation, which did not contain any agrowaste. While most of the biomass types tested (i.e., sugarcane bagasse, wheat bran, and rice straw) negatively impacted laccase production, enhancement was observed for wheat straw and cotton stalk. Particularly, wheat straw showed an even stronger positive effect than copper sulfate and was found to be one of the three main factors (including time and pH) that most positively affected the production of laccase.

FTIR analysis of the biodegraded biomass from experimental run 4 of RSM Plackett–Burman design was performed against two controls, pristine WS and CS. The aromatic stretching peak at 1610 cm^−1^ disappeared, indicating the complete removal of lignin by laccase.

## References

[CR1] Adapa P, Schonenau L, Canam T, Dumonceaux T (2011) Quantitative analysis of lignocellulosic components of non-treated and steam exploded barley, canola, oat and wheat straw using Fourier transform infrared spectroscopy. J Agric Sci Tech 177

[CR2] Atalla MM, Zeinab HK, Eman RH, Amani AY, Abeer AAEA (2013) Characterization and kinetic properties of the purified *Trematosphaeria**mangrovei* laccase enzyme. Saudi J Biol Sci 20:373–381 24235874 10.1016/j.sjbs.2013.04.001 PMC3824134

[CR3] Banci L, Ciofi-Baffoni S, Tien M (1999) Lignin and Mn peroxidase-catalyzed oxidation of phenolic lignin oligomers. Biochem 38:3205–3210 10074376 10.1021/bi982139g

[CR4] Benfield G, Bocks SM, Bromley K, Brown B (1964) Studies of fungal and plant laccases. Phytochem 3:79–88

[CR5] Bera SP, Tank SJ (2021) Microbial degradation of Procion Red by *Pseudomonas **stutzeri*. Sci Rep 11:3075 33542307 10.1038/s41598-021-82494-9 PMC7862368

[CR6] Ding Z, Peng L, Chen Y, Zhang L, Shi G, Zhang K (2012) Production and characterization of thermostable laccase from the mushroom, *Ganoderma**lucidum*, using submerged fermentation. Afr J Microbiol Res 6:1147–1157

[CR7] Gomaa OM, Momtaz OA (2015) Copper induction and differential expression of laccase in *Aspergillus **flavus*. Braz J Microbiol 46:285–292 26221119 10.1590/S1517-838246120120118 PMC4512072

[CR8] Hakulinen N, Rouvinen J (2015) Three-dimensional structures of laccases. Cell Mol Life Sci 72:857–868 25586561 10.1007/s00018-014-1827-5 PMC11113281

[CR9] Hasan S, Anwar Z, Khalid W, Afzal F, Zafar M, Ali U, Refai MY, Afifi M, AL-Farga A, Aljobair MO (2023) Laccase production from local biomass using solid state fermentation. Ferment 9:179

[CR10] Hoegger PJ, Kilaru S, James TY, Thacker JR, Kües U (2006) Phylogenetic comparison and classification of laccase and related multicopper oxidase protein sequences. FEBS J 273:2308–2326 16650005 10.1111/j.1742-4658.2006.05247.x

[CR11] Iram A, Berenjian A, Demirci A (2021) A review on the utilization of lignin as a fermentation substrate to produce lignin-modifying enzymes and other value-added products. Mol 26:2960 10.3390/molecules26102960 PMC8156730 34065753

[CR12] Joshi AU, Hinsu AT, Kotadiya RJ, Rank JK, Andharia KN, Kothari RK (2020) Decolorization and biodegradation of textile di-azo dye Acid Blue 113 by *Pseudomonas stutzeri* AK6. 3 Biotech 10:214 32351872 10.1007/s13205-020-02205-5 PMC7182651

[CR13] Kameshwar AKS, Qin W (2017) Qualitative and quantitative methods for isolation and characterization of lignin-modifying enzymes secreted by microorganisms. BioEnergy Res 10:248–266

[CR14] Kirk TK, Farrell RL (1987) Enzymatic “combustion”: the microbial degradation of lignin. Ann Rev Microbiol 41:465–501 3318677 10.1146/annurev.mi.41.100187.002341

[CR15] Kuppusamy S, Sethurajan M, Kadarkarai M, Aruliah RJ (2017) Biodecolourization of textile dyes by novel, indigenous *Pseudomonas **stutzeri* MN1 and *Acinetobacter **baumannii* MN3. J Environ Chem Eng 5:716–724

[CR16] Lau SKP, Teng JLL, Ho C.-C, Woo PCY (2015) Chapter 12 - gene amplification and sequencing for bacterial identification. In “Methods in microbiology” (Sails A and Tang Y-W, eds.), Vol. 42, pp. 433–464. Academic Press

[CR17] Niladevi KN, Sukumaran RK, Jacob N, Anisha G, Prema P (2009) Optimization of laccase production from a novel strain—*Streptomyces **psammoticus* using response surface methodology. Microbiol Res 164:105–113 17207981 10.1016/j.micres.2006.10.006

[CR18] Parthipan P, Elumalai P, Sathishkumar K, Sabarinathan D, Murugan K, Benelli G, Rajasekar A (2017) Biosurfactant and enzyme mediated crude oil degradation by *Pseudomonas stutzeri* NA3 and *Acinetobacter baumannii* MN3. 3 Biotech 7:1–17 10.1007/s13205-017-0902-7 PMC5545984 28794933

[CR19] Quintanar L, Yoon J, Aznar CP, Palmer AE, Andersson KK, Britt RD, Solomon EI (2005) Spectroscopic and electronic structure studies of the trinuclear Cu cluster active site of the multicopper oxidase laccase: nature of its coordination unsaturation. J Am Chem Soc 127:13832–13845 16201804 10.1021/ja0421405

[CR20] Rajeswari M, Vennila K, and Bhuvaneswari V (2015) Optimization of laccase production media by *Bacilllus cereus* TSS1 using Box-Behnken design. Magnesium 1:0

[CR21] Reiss R, Ihssen J, Richter M, Eichhorn E, Schilling B, Thöny-Meyer L (2013) Laccase versus laccase-like multi-copper oxidase: a comparative study of similar enzymes with diverse substrate spectra. PLoS One 8:e65633 23755261 10.1371/journal.pone.0065633 PMC3670849

[CR22] Shinde R, Wasule D, Anjali MG, Parlawar N, Manali SS, Patle KP, Khating HK, Meshram A, Ghutake SR, Khan RMR (2020) Hyper-production of laccase a green catalyst by RS2 (*Pseudomonas **stutzeri* CW202) through mutagenesis. Int J Chem Stud 8:04–08

[CR23] Singh G, Ahuja N, Sharma P, Capalash N (2009) Response surface methodology for the optimized production of an alkalophilic laccase from gamma-proteobacterium JB. BioResources 4:544–553

[CR24] Vignesh R, Madhusudhanan J, Gandhiraj V, Deepika RC (2016) Screening and characterization of laccase from fungal isolates. IRJET 0056–0072

[CR26] Zeng J, Singh D, Chen S (2011) Biological pretreatment of wheat straw by *Phanerochaete**chrysosporium* supplemented with inorganic salts. Bioresour Technol 102:3206–3214 21111608 10.1016/j.biortech.2010.11.008

